# A Rare Location and Presentation of Pheochromocytoma

**DOI:** 10.1089/cren.2018.0025

**Published:** 2018-07-01

**Authors:** Kevin Kaulanjan, Pascal Blanchet, Laurent Brureau

**Affiliations:** ^1^ Service d'urologie et de transplantation rénale, CHU de Point-à-Pitre, Pointe-à-Pitre, Guadeloupe, France.; ^2^ INSERM U 1085, IRSET, Pointe-à-Pitre, Guadeloupe, France.

**Keywords:** pheochromocytoma, bladder cancer, rare tumor, transurethral bladder resection, laparoscopic robot-assisted surgery

## Abstract

***Background:*** Pheochromocytomas typically are diagnosed in the adrenal gland and from the sympathetic nervous system. Bladder pheochromocytoma is a rare location for this tumor.

***Case Presentation:*** We describe a 67-year-old Afro Caribbean woman referred to our hospital for an asymptomatic bladder tumor. Preliminary transurethral resection revealed bladder pheochromocytoma. After a comprehensive endocrine evaluation, we performed a robot-assisted laparoscopic partial cystectomy with ureteral reimplantation.

***Conclusion:*** We present a rare case of bladder pheochromocytoma treated effectively with minimally invasive techniques. When confronted with a solid bladder mass, apart from the more common urothelial malignancies, a differential diagnosis of bladder pheochromocytoma should also be considered.

## Introduction

Extra-adrenal pheochromocytomas located in the urinary bladder are rare occurrences and are seen in less than 1% of cases. We herein report a totally atypical presentation of bladder pheochromocytoma.

## Presentation of the Case

### Clinical history

A 67-year-old Afro Caribbean asymptomatic woman came to the urology outpatient clinic because of ultrasonographic finding of a bladder mass. In her medical history, we found diabetes mellitus, hypothyroidism, and well-controlled high blood pressure. She had no family history of pheochromocytoma or paraganglioma. She denied any low urinary tract symptom such as hematuria, dysuria, polyuria, or incontinence. The general condition was preserved, without appetite disturbance or weight loss.

### Physical and para clinical examinations

**FIG. 1.**
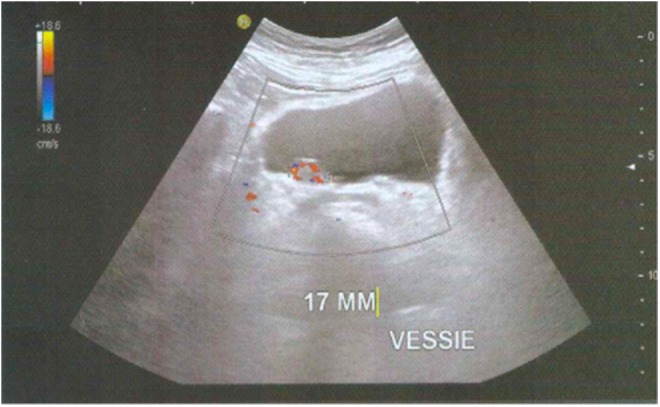
Ultra sonogram of the urinary bladder showing a bladder nodule.

The patient had 135/88 mm Hg of blood pressure and no fever. Abdominal evaluation did not reveal any palpable mass nor tenderness. Palpation of thyroid and lymphatic areas were unremarkable. Digital rectal and vaginal examinations were normal. Likewise, the urinary output test showed a good flow. The ultrasound, however, revealed a 17 mm bladder mass on the right posterolateral wall (Fig. 1).

During cystoscopy procedure, we found a right periureteral submucosal lesion. We performed a transurethral resection of the lesion under general anesthesia. Because of the resection of the right ureteral orifice, we placed a Double-J stent. The blood pressure remained stable throughout the procedure and no variations were noted.

### Diagnosis and intervention

Histopathologic analysis revealed the diagnostic of extra-adrenal pheochromocytoma of the urinary bladder. Immunohistochemical stains were positive for chromogranin A, Synaptophysin, CD56, and S-100 protein. Both urinary and plasma metanephrine tests were normal.

**FIG. 2.**
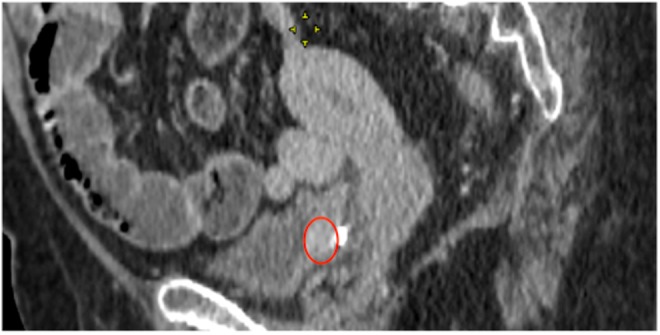
CT showing an enhancing mass (*red circle*) in the posterolateral right bladder wall.

A follow-up chest-abdomen-pelvis contrast-enhanced CT revealed the persistence of a 20 mm posterolateral bladder mass at the resection site (Fig. 2). There were no other suspicious extra-adrenal sites.

The diagnosis of nonsecreting paraganglioma of the urinary bladder was confirmed. A multidisciplinary team was convened and recommended to perform a partial cystectomy.

**FIG. 3.**
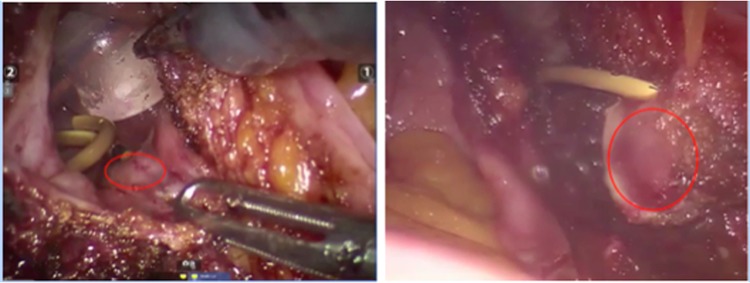
Per operative view before and after removal of the tumor (*red circles*).

After a comprehensive anesthetic evaluation, we performed a robot-assisted laparoscopic partial cystectomy with relocation of the right ureter. The patient was placed in the Trendelenburg position (25 degrees angulation). With an intraperitoneal approach, we identified the ureter and dissected it carefully up to the bladder insertion. A cystostomy was performed to visualize the tumor intravesically (Fig. 3). We excised the tumor and the ureteral orifice carefully with adequate margins to avoid residual tumor tissue. This was followed by an extravesical Lich-Gregoir ureteral reimplantation over a Double-J ureteral stent. We left a 14F indwelling Foley catheter. The blood loss was less than 150 mL. The postoperative course was uneventful. The patient was discharged after 2 days. After 10 days, a nurse at home removed the urinary catheter, and 6 weeks after, the Double-J stent was removed on an outpatient basis.

### Follow-up

**FIG. 4.**
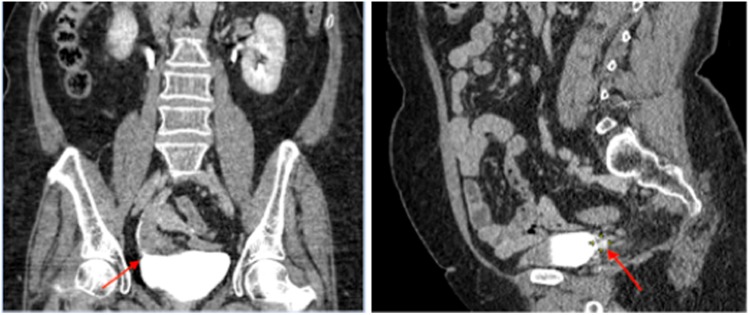
Coronal and sagittal view of CT after 12 months follow-up. *Arrows* show the new ureteral implantation.

The final pathology confirmed a 5 mm paraganglioma. No mitoses and no lymph vascular invasion were identified. Immunohistochemical staining was strongly positive for chromogranin A, synaptophysin, and S-100 protein. The surgical margins were free of tumor. After a 24-month follow-up period, the patient was asymptomatic. The CT of urinary tract found an unremarkable urinary excretion and no recurrence of the tumor (Fig. 4).

## Discussion

Extra-adrenal pheochromocytomas, also called paragangliomas, are neuroendocrine tumors that usually develop from the chromaffin cells. Thus, the tumor could be located between the base of the skull and the pelvis.

Pheochromocytomas of the urinary bladder are exceedingly rare tumors accounting for less than 0.05% of all bladder tumors and less than 1% of all pheochromocytomas. Paragangliomas are generally benign tumors, with only less than 10% being malignant.^1^ Most of these tumors are hormonally active, and secrete mainly noradrenaline (and rarely adrenaline), calcitonin and adrenocorticotropic hormone. Due to high catecholamine level, the common symptoms are micturition attacks, hypertensive crisis, hematuria, headache, or tachycardia triggered by urination. In our case, none of these symptoms was present. It was a nonfunctioning paraganglioma with an incidental finding. In fact, in addition to the absence of clinical signs, all endocrine tests, both serum and urine, were negative. When we performed the cystoscopy and transurethral resection of the bladder mass, we had no suspicion of this pathology. The absence of hypertensive crisis during the intervention is a further evidence of a nonsecreting tumor.

The diagnosis of pheochromocytoma of the urinary bladder is confirmed with a combination of specific symptoms, laboratory tests, and image investigations. It is extremely difficult to preoperatively diagnose an asymptomatic bladder paraganglioma. In our case, the bladder resection gave us the diagnosis. The chest–abdomen–pelvis CT was used to search other extra-adrenal sites. In fact, this investigation gives a better sensitivity than the metaiodobenzylguanidine scan (89% against 81%).^2^ Thus, in presence of submucosal mass of the bladder, the diagnosis of paraganglioma should be considered.

The treatment requires a partial or total cystectomy after an adequate anesthetic preparation to make the surgery safe.^3^ The laparoscopic robot-assisted surgery offers a safe way to realize this intervention with low blood loss and low postoperative complications compared to open surgery. This minimally invasive surgery offers possibility of enhanced recovery after surgery and anesthesia. Our case is unique for several reasons. First, many reviews of paragangliomas of the urinary bladder have been published but nonfunctioning paragangliomas are rarely reported. Second, to our knowledge, it is the second report of a robotic resection of a bladder pheochromocytoma with ureteric reimplantation.^4^

## Conclusion

In the presence of a solid mass in the bladder wall, a diagnosis of bladder pheochromocytoma should be considered, even with an atypical presentation. The laparoscopic robot-assisted surgery should be considered as a safe way to treat this pathology.

## Abbreviation Used

CT: computed tomography
